# Effectiveness and safety of everolimus treatment in patients with tuberous sclerosis complex in real-world clinical practice

**DOI:** 10.1186/s13023-023-02982-1

**Published:** 2023-12-02

**Authors:** Ine Cockerell, Jakob Christensen, Christina E. Hoei-Hansen, Lotte Holst, Mikkel Grenaa Frederiksen, Aart Imran Issa-Epe, Bård Nedregaard, Ragnar Solhoff, Ketil Heimdal, Cecilie Johannessen Landmark, Caroline Lund, Terje Nærland

**Affiliations:** 1 Department of Rare Disorders and Disabilities, National Centre for Rare Epilepsy-Related Disorders, Oslo University Hospital, Pb 4950, 0424 Nydalen, Oslo Norway; 2 Department of Clinical Medicine, Aarhus University, Aarhus, Denmark; 3 Department of Neurology, Aarhus University Hospital, Affiliated Member of the European Reference Network EpiCARE, Aarhus, Denmark; 4 Department of Clinical Medicine, Copenhagen University, Copenhagen, Denmark; 5 Department of Paediatrics, University Hospital Rigshospitalet, Copenhagen, Denmark; 6 Section of Abdominal Radiology, Department of Radiology, Oslo University Hospital, Oslo, Norway; 7 Section of Neuroradiology, Department of Radiology, Oslo University Hospital, Oslo, Norway; 8 Department of Neurology, Sørlandet Hospital, Arendal, Norway; 9 Department of Medical Genetics, Oslo University Hospital, Oslo, Norway; 10 Department of Pharmacy, Faculty of Health Sciences, Oslo Metropolitan University, Oslo, Norway; 11 The National Center for Epilepsy (SSE), Member of the ERN EpiCare, Oslo University Hospital, Oslo, Norway; 12 Section for Clinical Pharmacology, SSE, Department of Pharmacology, Oslo University Hospital, Oslo, Norway; 13 K.G. Jebsen Center for Neurodevelopmental Disorders, Institute of Clinical Medicine, University of Oslo, Oslo, Norway; 14 NevSom, Department of Rare Disorders and Disabilities, Oslo University Hospital, Oslo, Norway

**Keywords:** Tuberous sclerosis complex, mTOR inhibitor, Everolimus, Sirolimus, Subependymal giant cell astrocytoma, Renal angiomyolipoma, Epilepsy, Adverse events

## Abstract

**Background:**

The randomised double-blinded placebo-controlled EXIST-1–3 studies have showed everolimus effective with adverse effects reported as acceptable in treatment of symptoms in patients with tuberous sclerosis complex (TSC), although evidence of outcomes in clinical practice remains limited. This study aimed to investigate, in clinical practice, the effectiveness and safety of everolimus for epilepsy, renal angiomyolipoma (rAML), and subependymal giant cell astrocytoma (SEGA) in patients with TSC.

**Results:**

The study included 64 patients with TSC (median age: 19, range 0.9–54 years) receiving everolimus treatment (Norway: n = 35; Denmark: n = 29). Among 45 patients with epilepsy, 14 (31%) were responders experiencing ≥ 50% reduction in seizure frequency in the last 3 months of treatment compared with the last 3 months before treatment. Nineteen (42%) patients changed their anti-seizure medications (ASMs). Responders were more common among patients < 18 years (46%) than among patients ≥ 18 years (14%, *p* = 0.03). In 29 patients with rAML, everolimus reduced (≥ 30% decrease) and stabilized (< 20% increase, ≤ 30% decrease) longest diameter of rAML in 38% and 59%, respectively, after a mean treatment duration of 37 months. SEGA volume was reduced in three patients by 71%, 43%, and 48% after 39, 34, and 82 months. Adverse effects were reported in 61 of 64 patients (95%) after a median treatment duration of 31 months (range 0–106), with oral ulceration/stomatitis (63%) and upper respiratory tract infections (38%) being the most common. The most common laboratory abnormalities were increased cholesterol (41%), anaemia (30%), and leucopoenia (25%). Grade 3–4 adverse effects were reported in 36% of cases, and life-threatening conditions were reported in two patients. Nine patients discontinued everolimus treatment.

**Conclusions:**

Seizure reduction in this study sample was consistent with results from EXIST, but might be lower than expected, given that changes in concomitant ASMs are part of clinical practice. Seizure reduction was associated with younger age. As with EXIST, everolimus reduced or stabilised rAML size in most patients. SEGA volume was reduced in all three patients. Close follow-up is needed for this group, especially for children and patients who may not be able to report adverse effects.

**Supplementary Information:**

The online version contains supplementary material available at 10.1186/s13023-023-02982-1.

## Background

Tuberous sclerosis complex (TSC) is a rare autosomal dominant genetic disease caused by genetic variants in TSC1 [1] or TSC2 [2]. The genes code for the proteins hamartin [1] and tuberin [2] that inhibit the mammalian target of rapamycin (m-TOR) pathway, which controls cellular growth and metabolism [3, 4]. TSC is characterised by benign tumours in different organs [3, 4]. Epilepsy, neurocognitive deficits, and neuropsychiatric disorders, including autism [5], and lesions in the brain, skin, kidney, and lungs are common [3, 4]. Epilepsy, renal symptoms, and neuropsychiatric disorders are associated with the greatest morbidity and mortality [5–8]. Renal angiomyolipomas (rAML) are present in 48–70% of cases [6, 9–13], and carry a risk of spontaneous bleeding [11, 12, 14], impaired renal function [12] and end stage renal disease [15–17]. Subependymal giant cell astrocytomas (SEGA) in the brain have been described in up to 24% of cases and carry a risk of hydrocephalus requiring surgery or shunt placement [18]. Epilepsy is described in up to 93% [19], and up to 63% develop refractory epilepsy [20].

TSC treatment involves mTOR-inhibitors. These drugs block the mTOR-complex activation, reducing tumour growth, and offer a potential disease-modifying approach [21].

The safety and efficacy of the mTOR inhibitor everolimus was studied in a series of randomised, double-blinded, placebo-controlled trials (EXIST-1–3) for the treatment of SEGA, rAML, and epilepsy [22–24]. At least a 50% reduction in SEGA and rAML volume was achieved in 35% and 42% [22, 23] of treated patients, respectively. At least a 50% reduction in the frequency of epileptic seizures was achieved in 28% of patients with low (3–7 ng/mL) and 40% of patients with high (9–15 ng/mL) everolimus exposure [24]. In extension studies of EXIST 1–3, the safety profile was considered satisfactory, with adverse effects mainly classified as mild to moderate [25–27]. Nevertheless, the adverse effects of everolimus may affect quality of life, require dose reduction, or interrupt treatment [28], and severe life-threatening infections and deaths have been reported [27, 29]. Mouth ulcers, delayed wound healing, infections, and metabolic and haematological disturbances are the most common adverse effects [30].

Randomized controlled trials (RCT) are the most reliable design to investigate effects on interventions, [31, 32] and the most reliable source for treatment decisions [33]. Risk of bias is minimized by patient randomization, allocation and blinding, making it possible to conclude that the effect is caused by the intervention [31, 34].

However, the stringent trial settings in RCTs with highly selected patients and short follow up differ from the complexities in routine clinical practice [31, 35]. Studies from “real world” could give results that are more generalizable to routine clinical practice [31, 35] and add information needed to make treatment decisions [36].

Population-based studies investigating the effectiveness and safety of mTOR inhibitors in clinical practice are limited [37–46], and those available are partly inconsistent, indicating that there is still a knowledge gap in this field. For instance, reported frequency of adverse effects varied between 42 and 71% [37, 39, 41], and reported proportions of patients with ≥ 50% seizure reduction varied between 33 and 78% [39, 41, 47].

The aim of this study was to investigate the effectiveness and safety of treatment with everolimus in patients with TSC in real-world clinical practice.

## Results

### Patient characteristics

Table 1 summarises the patient characteristics. Sixty-four patients were included (Norway: n = 35; Denmark: n = 29). Most patients (63%) were female. Four patients were treated for more than one indication (AML/SEGA: 2, AML/LAM: 1, epilepsy/AML/LAM: 1). A large proportion of patients had multiple TSC-related symptoms in addition to the indication they were treated for. In total, 61% of patients had a TSC2 pathogenic variant, and 5% had a TSC1 pathogenic variant; 34% of those with TSC had no available data on genetic testing or had not been tested.

**Table 1:** Patient characteristics

	All indications (n = 64)	Epilepsy indication (n = 28)	Renal AML indication (n = 29)	SEGA and LAM indications (n = 5)
Age at start of treatment				
Mean ± SD (CI)	20 ± 13.98 (16.5–23.5)	11 ± 12.16 (6.6–16.1)	27 ± 10 (22.9–30.6)	21 ± 13.25 (4.5–37)
Median (min–max)	19 (0.9–54)	6 (09–44)	26 (8–54)	22 (6–37)
Age start treatment (grouped)				
< 6 years, n (%)	16 (25)	15 (54)		1 (20)
6–17 years, n (%)	13 (20)	6 (21)	6 (21)	1 (20)
≥ 18 years, n (%)	35/(55)	7 (25)	23 (79)	3 (60)
Months of follow up				
Mean ± SD (CI)	37/25 (30–43)	27/22/(18–35)	37 ± 4.74 (26–46)	52 ± 2 6.95 (19–85)
Median (min–max)	31 (0–106)	19 (3–84)	38 (5–96)	39 (25–82)
Intellectual disability, n (%)	43 (67)	22 (79)	19 (70)	5 (100)
Autism spectrum disorder, n (%)	25 (39)	13 (46)	10 (35)	2 (40)
Male, n (%)	24 (37.5)	14 (50)	9 (31)	2 (40)
Female, n (%)	40 (62.5)	14 (50)	20 (69)	3 (60)
Mutation				
TSC1, n (%)	3 (5)	2 (7)		1 (20)
TSC2, n (%)	39 (61)	21 (75)	17 (58)	2 (40)
No mutation identified, n (%)	10 (15)	4 (14)	6 (21)	
Not tested/missing, n (%)	12 (19)	1 (4)	6 (21)	2 (40)
Renal AML, n (%)	47 (73)	12 (43)	29 (100)	5 (100)
SEGA lesions, n (%)	17 (27)	7 (25)	5 (17)	5 (100)
Epilepsy, n (%)	45 (70)	28 (100)	14 (48)	4 (40)
Lung manifestations, n (%)	6 (9)	1 (4)	6 (21)	2 (40)
Facial angiofibroma, n (%)	47 (73)	15 (54)	27 (93)	2 (40)

### Epilepsy

Twenty-eight of 64 patients (44%) were treated with everolimus for epilepsy as the primary indication (epilepsy indication group). Further, 17 (27%) had epilepsy but were primarily treated with everolimus for other indications (other indication group). Thus, in total, there were 45 patients in the entire epilepsy group (70% of the study’s patient sample).

Seizure frequency per month at the start of treatment was higher in patients treated for epilepsy indication (mean/median: 32/28, SD: 36.6, range 0.25–175) than in patients treated for other indications (mean/median: 3.2/1.5, SD 4.12, range 0–14), and there were more patients with ≥ 3 seizure types (43 vs. 24%) and ≥ 3 ASMs (50 vs. 35%) at the start of treatment among patients treated for epilepsy than in patients treated for other indications. At least 50% seizure reduction occurred in 31% of the entire epilepsy group and was quite similar in both groups (Table 2). Any seizure reduction and at least 30% seizure reduction occurred in 68% and 44% of the entire epilepsy group (Additional file 1: Tables S1 and S2).

**Table 2:** Seizure reduction (≥ /< 50%) and related factors

		Entire epilepsy group (n = 45)			Other indications group (n = 17)			Epilepsy indication group (n = 28)	
		≥ 50% seizure reduction n/N( %)	< 50% seizure reduction n/N( %)		≥ 50% seizure reduction n/N( %)	< 50% seizure reduction n/N( %)		≥ 50% seizure reduction n/N(%)	< 50% seizure reduction n/N (%)
All		14/45 (31)	30/45 (69)*		5/17 (29)	12/17 (71)		9/28 (32)	19/28 (68)*
Norway		4/26 (15)	22/26 (85)		1/7 (14)	6/7 (86)		3/19 (16)	16/19 (84)
Denmark		10/19 (53)	9/19 (47)		4/9 (44)	6/9 (66)		6/9 (67)	3/9 (33)
≥ 3 seizure types before treatment	Yes	4/10 (40)	6/10 (60)		4/13 (39)	9/13 (31)		3/6 (50)	3/6 (50)
	No	10/35 (29)	25/35 (71)		1/4 (25)	3/4 (75)		6/22 (27)	16/22 (73)
GTK before treatment	Yes	6/22 (27)	16/22 (73)		3/10 (30)	7/10 (70)		3/12 (25)	9/12 (75)
	No	8/23 (65)	15/23 (65)		2/7 (29)	5/7 (71)		6/16 (37.5)	10/16 (62.5)
≥ 3 ASMs at start of treatment	Yes	6/20 (30)	14/20 (70)		3/6 (50)	3/6 (50)		6/14 (42)	8/14 (58)
	No	8/25 (32)	17/25 (68)		2/11 (18)	9/11 (82)		3/14 (21)	11/14 (79)
Median weekly seizure**	< 7	5/15 (33)	10/15 (67)	< 1.5	1/5 (20)	4/5 (80)	< 28	4/8 (50)	9/18/(50)
Frequency before treatment	≥ 7	9/26 (35)	17/26 (65)	≥ 1.5	3/9 (33)	6/9 (67)	≥ 28	4/8 (50)	9/18/(50)
Age at start of treatment	< 18	11/24 (46)	13/24 (54)*		2/3 (67)	1/3 (33)		9/21 (43)	12/21 (57)
	≥ 18	3/21 (14)	18/21 (86)		3/14 (21)	11/14 (79)		0/7 (0)	7/7 (100)
Major change in ASMs	No	7/26 (27)	19/26 (73)		3/10 (30)	7/10 (70)		4/16 (25)	12/16 (75)
	Yes	7/19 (37)	12/19 (63)		2/7 (29)	5/7 (71)		5/12 (42)	7/12 (58)

*Significant difference

The proportions of patients with ≥ 50% (Table 2) and ≥ 30% seizure reduction were higher in Denmark (Additional file 1: Table S2), but only significantly higher in the proportions with ≥ 50% seizure reduction. The proportion with any seizure reduction was similar in both countries (Additional file 1: Table S1). Age at start of treatment for epilepsy indication was lower in Denmark (mean: 9 years/median: 3 years) than in Norway (mean: 12 years/median: 7 years), but the difference were not significantly lower.

**Fig. 1:**
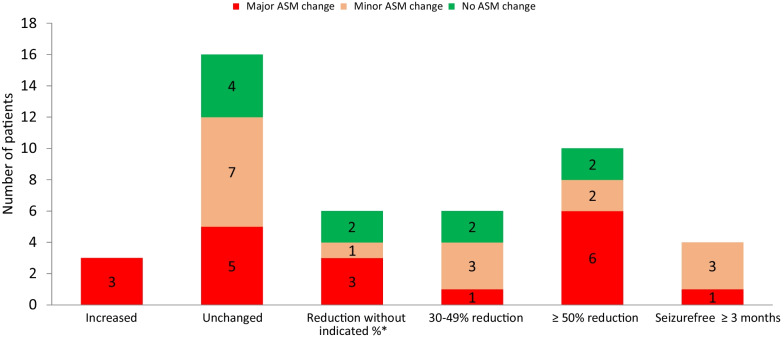
Change in seizure frequency in patients with epilepsy (n = 45). *Clinically relevant reduction without a given percentage (when percentage change was not available)

Figure 1 illustrates the change in seizure frequency and any ASM changes in the entire epilepsy group. ASM changes were described as none in 10, minor in 16, and major in 19 patients in the whole epilepsy group, and as none in 7, minor in 9, and major in 12 patients in the epilepsy indication group.

Forty-four (98%) had exact information about the number of ASMs during treatment. The number of ASMs decreased from baseline in 6 patients, increased in 10 patients in the entire epilepsy group, decreased in 5 patients, and increased in 6 patients in the epilepsy indication group. In the patients with ≥ 50% reduction in seizure frequency, one started vagus stimulation treatment, one changed the vagus stimulator, and one had epilepsy surgery during the treatment period.

Seizure reduction was associated with younger age. The proportions of patients with ≥ 50%, ≥ 30%, and any seizure reduction were significantly higher in patients < 18 years of age in the entire epilepsy group (Table 2, Additional file 1: Tables S1 and S2). Five patients started treatment before 2 years of age. Of these, ≥ 50% reduction in seizure frequency occurred in two patients, and ≥ 30%, no change, and increase in seizure frequency in one each of the other three patients.

Seizure reduction was not associated with the number of seizure types, focal to bilateral tonic clonic seizure, seizure frequency, number of ASMs, or major changes in the use of ASMs (Table 2, Additional file 1: Tables S1 and S2). A total of 25 patients in the epilepsy indication group and 36 patients in the entire epilepsy group had at least three measurements of serum concentration. The mean serum C/D ratio was not significantly different (*p* = 0.73) in those with ≥ 50% seizure reduction compared to those with < 50% seizure reduction in the epilepsy indication group (median 1.24 vs. 1.35) or the epilepsy group (1.09 vs. 1.1).

### Renal angiomyolipoma

**Fig. 2:**
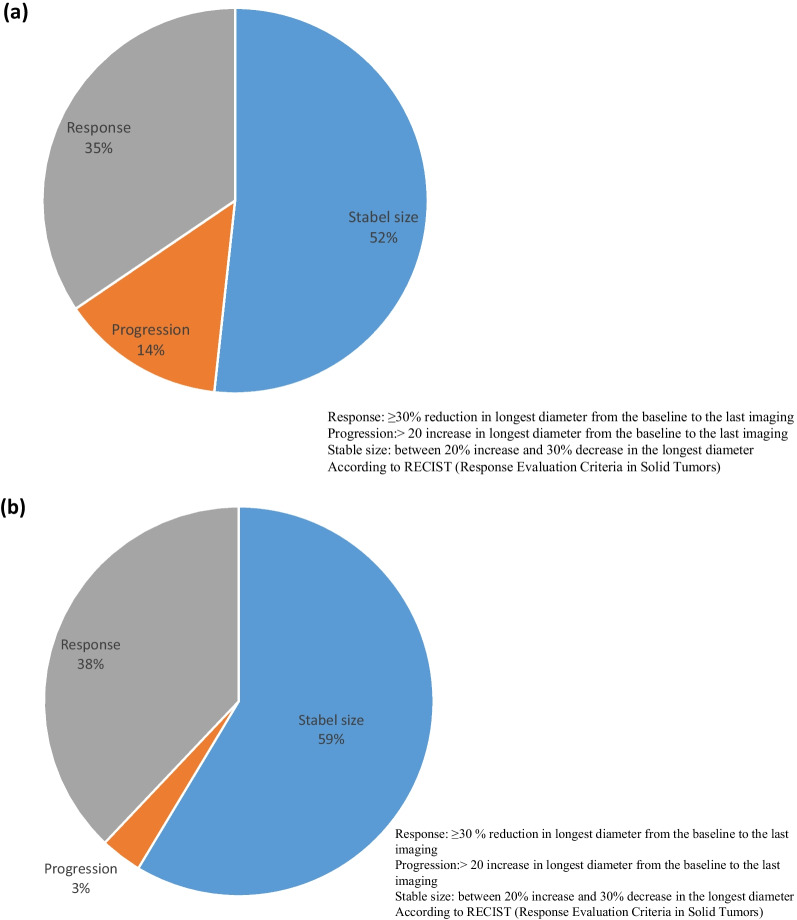
**a** Change in size of largest rAML from the baseline to the last imaging (n = 29). **b** Mean diameter change in largest rAML size in both kidneys from the baseline to the last imaging (n = 29)

Of all 64 included patients, 35 (55%) were treated for rAML, and 29 (45%) had rAML over 1 cm and imaging available. Response (≥ 30% decrease of LD) occurred in 35% when the change in LD was measured in the largest rAML and in 38% when the mean change was measured in the largest rAML in both kidneys. Stable size (< 20% increase, < 30% decrease in LD) occurred in 52% and 59% of the patients, respectively. Progression (> 20% increase in LD) occurred in 1 (7%) and 4 (14%) of the 29 patients (Fig. 2).

Table 3 summarizes the renal characteristics at baseline and at the last imaging. The percentage of patients with an rAML with a diameter > 4 cm decreased from 75% at baseline to 55% at last imaging, and the percentage of those with an rAML with a diameter > 6 cm decreased from 31% at baseline to 24%at last imaging The number of rAML with a diameter > 1 cm was similar at baseline and at the last imaging. Renal morphology was not recognisable for two patients at baseline and for two patients at the last imaging. Eight patients (28%) with rAML had other renal symptoms or interventions, including five patients (17%) with renal haemorrhage before treatment. The patients treated with embolisation and unilateral nephrectomy before treatment had renal haemorrhage (Table 3).

**Table 3:** Renal disease characteristics at baseline and at last imaging

	Baseline	Last imaging
Largest diameter of largest rAML in intervals (n = 29)		
< 40 mm, n (%)	7 (24)	13 (45)
40–60 mm, n (%)	9 (31)	7 (24)
> 60 mm, n (%)	13 (45)	9 (31)
Number of rAML > 1 cm (n = 28)		
< 5, n (%)	4 (14)	5 (17)
Between 5–20, n (%)	5 (17)	4 (14)
Between 5–20 in each kidney, n (%)	3 (10)	3 (10)
> 40, n (%)	16 (55)	16 (55)
Growth in LD last year before treatment (n = 9)**		
< 0.25 cm, n (%)	2 (22)	–
≥ 0.25 cm, n (%)	7 (79)	–
Renal morphology		
Normal left/right kidney, n (%)	19 (68)/21 (72)	21 (75)/19 (68)
Recognizable left/right kidney, n (%)	7 (25)/6 (21)	6 (21)/8 (29)
Not recognizable left/right kidney, n (%)	2 (7)/2 (7)	2 (7)/2 (7)
Renal symptoms or interventions		
Haemorrhage	5 (17)	1 (4)
Embolisation	1 (4)	
Cryotherapy	2 (7)	
Unilateral nephrectomy	1 (4)	
Cancer	1 (4)	
Bilateral cysts, n (%)	20 (69)	20 (69)
Renal symptoms or interventions		
Haemorrhage	5 (17)	1 (4)
Embolisation	1 (4)	
Cryotherapy	2 (7)	
Unilateral nephrectomy	1 (4)	
Cancer	1 (4)	
Bilateral cysts, n (%)	20 (69)	20 (69)

**Patients with information

Nineteen patients had at least three measurements of the serum concentration of everolimus. C/D-ratio (mean serum concentration/dose) was not significantly different (*p* = 0.57) in responders compared to non-responders (1.06 vs. 1.35) when the mean change in rAML was measured in both kidneys. It was higher (median 1.35 vs. 0.72), but not significantly different (*p* = 0.32) in responders compared to non-responders when the change in rAML was measured in the largest rAML. The quality of the imaging was generally described as good.

### SEGA

Five patients were treated with everolimus for SEGA. For two of them, the first available imaging were taken 5 and 7 months after start of treatment, making only three available for evaluation of the effects. The SEGA volume of the largest SEGA decreased from 111.52 mm^3^, 57.34 mm^3^, and 28.6 mm^3^ at baseline to 32.64 mm^3^, 32.60 mm^3^ and 14.83 mm^3^ at the last imaging. The reduction in volume was 71%, 43%, and 48% after 39, 34, and 82 months of treatment, respectively.

### Safety

Sixty-one of the 64 (95%) patients reported clinical adverse effects after follow-up periods of 1–12 months (n = 10), 13–24 months (n = 13), 25–36 months (n = 18), and > 36 months (n = 23) (Table 4). Infectious episodes were the most common adverse effects. Oral ulceration/stomatitis (63%), upper respiratory tract infection (38%), and rash (27%) were the most frequent adverse effects during the entire treatment period. In the first and second years, pyrexia (16% and 17%) was the most frequent clinical adverse effect after stomatitis/oral ulceration (44% and 33%) and upper respiratory tract infection (30% and 20%). After the second year, skin infection (including erythema nodusum), pneumonia, and rash (all 12%) were the most frequent clinical adverse effects after stomatitis/oral ulceration (42%). Other adverse effects reported during the entire treatment period were fatigue (22%) and amenorrhea/irregular menses (29% of female patients over 20 years). Diabetes was reported in one patient.

**Table 4:** Adverse effects with dose modification over time during the treatment period

	1st year All grades n = 64 n/%	Grade 3–4 n = 64 n/%	2st years All grades n = 54 n/%	Grade 3–4 n = 54	After 2sd years All grades n = 41 n/%	Grade 3–4 n = 41 n/%	Whole period (all grades and non-graded) n = 64 n/%	Grade 3–4 n/N/%	Dose modification whole period n = 64 n/N/%
Infections									
Stomatitis/mouth ulceration	28/44	1/2	18/33		17/42		40/63	2/35/6	10/40/25
Upper respiratory tract infection	19/30	1/2	11/20		3/7		24/38		1/24/4
Rash	9/14		2/4		5/12		17/27		3/17/18
Pyrexia	10/16	4/6	9/17	3/6	1/2	1/2	13/20	4/12/33	4/13/31
Skin infection included erythema nodusum	7/11	1/2		1/2	5/12	1/2	11/17	3/12/25	4/11/37
Nausea/vomiting	7/11		1/2		3/7		9/14	1/9/11	2/9/22
Diarrhoea	6/10	1/2	2/4		1/2		9/14		1/9/11
Dermatitis acne	4/6		6/11		3/7		8/13		2/8/25
Pneumonia	5/8	5/8	3/6	2/4	5/12	4/10	8/13	8/8/100	5/8/63
Gastroenteritis	1/2		3/10		1/2		7/11		3/7/43
Urinary tract and/or pyelonephritis infections	3/5		4/7		2/5		6/9	1/6/17	2/6/33
Otitis	4/6		1/2		2/5		5/8		
Genital infection*					1/2		3/5		
Immune deficiency	1/2	1/2	2/4	2/4	1/2	1/2	2/3	2/64/3	
Abscess	1/2						1/2		
Infected polyp					1/2		1/2		
Mononucleosis	1/2						1/2		1/1/100
Hepatitis B virus			1/2				1/2		
Appendicitis	1/2						1/2		
Acute encephalitis	1/2						1/2	1/64/2	1/1/100
Chronic osteomyelitis			1/2		1/2	1/2	1/2	1/64/2	
Other adverse effects									
Tiredness	9/14	1/2	4/7	1/2	5/12		14/22	2/64/14	1/14/7
Pruritus			1/2				7/11		
Amenorrhea or irregular menses n/N/%	4/21/19		1/21/5				6/21/29		
Cysts **	2/4		3/6	2/4			6/9	2/64/14	
Constipation	3/5		1/2				5/8		
Headache							2/3		
Diabetes mellitus	1/2						1/2		1/1/100
Cerebral oedema	1/2	1/2					1/2	1/64/2	1/1/100
Laboratory abnormalities									
Any lab abnormality***	35/55		29/4		16/39		46/72		
Increased cholesterol	25/39		22/41		18/44		26/41		2/26/8
Hypertriglyceridemia	13/20		8/15		8/20		19/30		
Neutropenia	7/11		6/11	2/4	4/10	3/7	7/11	4/64/6	1/7/14
Leucopoenia	13/25	1/2	7/13	1/2	6/15	1/2	16/25	2/64/4	3/16/19
Anaemia	12/19		6/11		5/12		17/27		
Hyperglycaemia	1/2	1/2	2/4		3/7		4/6	1/64/2	1/4/25
Hypophosphatemia	5/8		1/2		2/5		6/9		1/6/17
Thrombocytopenia	4/7	1/2	2/4				4/6	1/64/2	1/4/25
ASAT	1/2						1/2		
ALAT	2/3	1/2					2/3	1/64/2	1/2/50
GGT	2/3		2/4		1/2		3/5		

*Genital infection (vaginal infection n = 2, testes infection n = 1)

**Cysts: (ovarian, clitoris, pilonidal)

***According to the National Cancer Institute Common Terminology Criteria version 5

Frequency of the most common adverse effects (stomatitis/oral ulceration, upper respiratory tract infections, fever, and skin infections) declined by ≥ 10% over time. However, a minority of the patients had stomatitis/mouth ulceration (30%) or upper respiratory tract infections (25%) regularly or almost continuously (13%). Hypercholesterolemia (41%), anaemia (30%), and leucopoenia (25%) were the most frequent laboratory abnormalities during the entire treatment period and in the first, second, and after the second treatment year. Hypercholesterolemia was mostly grade 1; only four patients had grade 2 (max cholesterol 10.2 mmol/L). Four patients required statin treatment.

Table 4 describes the types of adverse effects, severity, and dose modifications. Adverse effects ≥ grade 3 occurred in 36% of the patients during the entire treatment period. The most frequent adverse effect ≥ grade 3 was pneumonia, which occurred in 13% of the patients. Patients with adverse effects ≥ grade 3 were younger than patients without adverse effects ≥ grade 3 during the entire treatment period (*p*: 0.02, Md: 12 vs. 23 years), in the first year (*p* = 0.02, median: 8 vs. 20 years), and after the second year (*p* = 0.03, median: 15 vs. 26 years).

Fifty percent required dose modifications due to adverse effects. Stomatitis/oral ulceration was the most frequent cause of dose modification. Eighteen patients with symptomatic stomatitis/oral ulceration had no dose modifications.

Hospitalisation or prolongation of hospitalisation was required in 34% of the sample, and one patient was diagnosed with immunodeficiency disorder and was hospitalised 10 times during 33 months of treatment. The occurrence of hospitalisation decreased after treatment with immunoglobulin for this patient.

Nine patients (14%) discontinued treatment; four due to adverse effects (leukopenia, neutropenia and COVID-19 infection, cerebral oedema, oral ulceration), three due to both adverse effects and loss of effect (infections in two, infection risk due to COVID-19 pandemic in one, epilepsy in two, and rAML in one), and two due to loss of effectiveness (epilepsy).

The median duration of treatment was 15 months (range 0–28, mean 13, SD: 7.9, 95% CI 6.9–19.1). The frequency of adverse effects and adverse effects ≥ grade 3 during the entire treatment period was similar in Norway and Denmark [Norway: 34/35 (97%) vs. Denmark: 27/29 (93%), Norway 14/35 (40%) vs. Denmark 9/29 (31%), respectively].

## Discussion

In this study, we demonstrated the effectiveness and safety aspects of treatment with everolimus in 64 patients with TSC in Norway and Denmark. This is one of few unselected, population-based studies from clinical practice in countries with similar health care systems, characterised by high-quality follow-up and equal access to health care. In the following sections, we compare the outcome measures with results from the randomised, double-blinded, placebo-controlled EXIST studies [22–26] and other clinical studies.

### Epilepsy effectiveness

The effectiveness of epilepsy treatment with everolimus, defined as at least 50% seizure reduction, was observed in one-third of patients with epilepsy in this study. This proportion is similar to the patients treated with a low dose of everolimus in EXIST-3 [24], and similar to another randomised, placebo-controlled trial [48].

This finding might be lower than expected, since most patients also changed ASMs during the study period. Interactions due to concomitant use of enzyme inducers could explain some of the changes, as CYP3A4-mediated metabolism of everolimus is affected by drugs such as carbamazepine, phenytoin, and phenobarbital [49, 50]. However, only six patients changed an enzyme-inducing drug.

In contrast to RCTs studies, it is difficult to know if the seizure frequency reduction is caused by the intervention alone in real world studies [31, 34].

In our study, the proportion of patients with ≥ 50% seizure reduction was higher in Denmark than in Norway.

The use of everolimus for epilepsy is restricted in Denmark, to ensure sufficient effect of everolimus, which is an expensive drug with potentially serious adverse effects. Thirty-three and fifty percent seizure reduction, good cooperation, and tolerable adverse effects are required after 4 and 12 months, respectively, to continue treatment. To fulfil these requirements, Danish patients submit seizure diaries for review, and the treating physician submits an evaluation to the Danish Medicines Authorities for documentation after the first treatment year. This is not required in Norway.

The observed variability in effectiveness between Norway and Denmark may not reflect a real discrepancy in efficacy, but may be influenced by the requirement to fulfill efficacy criteria in Denmark.

Another possible explanation is that patients treated for epilepsy indication were younger at start of treatment in Denmark than in Norway and although the difference were not significant, it is possible that also influenced the difference. In addition, there was not difference in patients with less than 50% reduction between Norway and Denmark.

The proportions of patients who reported ≥ 50% seizure reduction varied between 33 and 78% [39, 41, 47] in other clinical studies, possibly due to differences in inclusion and selection criteria. The study with the best effectiveness included participants without refractory epilepsy; all were children, and almost half of them were under 2 years [47]. This study reported > 50% seizure reduction in > 90% of 47 children aged less than 2 years treated with sirolimus [47].

Patients who started treatment before 2 years did not report a better effect in our study, although seizure reduction was associated with younger age, as also reported previously [27].

The number of ASMs did not decrease under everolimus treatment in our study. The different safety profiles of everolimus compared to other ASMs, gives a higher and different adverse effect load for patients treated with everolimus for epilepsy.

In contrast to the results from EXIST-3, which reported higher odds for response in patients treated with high exposure [24], seizure reduction was not associated with the calculated C/D-ratio in our study. This might be due to quite few participants, lack of data in some patients, less use of concomitant enzyme inducers among other antiseizure medications and extensive physiological and pharmacokinetic variability and tolerability between patients.

However, if the efficacy of everolimus is not dose dependent, as it seems in some other studies [38, 39], this could suggest that treatment with lower doses could be sufficiently efficacious and associated with fewer adverse effects. More studies are needed to investigate this.

No growth or a small reduction in rAML and SEGA lesions might be sufficient to prevent symptoms, whereas a small reduction in seizure frequency might not be as clinically relevant. However, a slight reduction in seizure frequency may make a difference in everyday life for persons with epilepsy [51], indicating that outcome measures ≤ 50% seizure frequency reduction could be clinical relevant, although a slight reduction in seizure frequency is a less reliable outcome measure. Seizure free days are a novel outcome measure [52] that could be considered in future studies.

A reasonable effect with regard to seizure reduction and tolerable adverse effects, as requested in Denmark, could, in general, be recommended to continue treatment for the epilepsy indication.

### RAML effectiveness

About one-third of our patients were responders (> 30% reduction of LD) versus 42% in EXIST-2 [23] and 58% in the final results of EXIST-2 [25]. These results are only partly comparable since the outcome measures in EXIST-2 differed from our study. EXIST-2 measured proportions of patients with ≥ 50% reduction in sum of volumes of all target angiomyolipomas (≥ 1 cm in LD) and measured change from baseline to best percentage change during treatment [53]. Our study measured proportions of patients with ≥ 30% reduction of LD of largest rAML and mean change of largest LD in both kidneys, and measured change from baseline to last imaging.

Volume measurement was not feasible for various reasons; it was too time-consuming, the automated method used for volume measurement did not capture accurate volume assessment, and some images were only taken in single sections. The change in size of rAML was measured according to Response Evaluation Criteria in Solid Tumors (RECIST) [54] because it is equivalent to volume measurement. Overall, 76% of the patients with rAML had at least a 10% reduction in the rAML size. In EXIST-2, volume reduction was described in 97% of the cohort [25]. In other clinical studies, this varied between 64 and 98% [41, 43, 46]. No renal bleeding was reported in our study, although a renal haemorrhage of older date was described on routine magnetic resonance imaging in one patient. No renal bleedings ≥ grade 2 were described during everolimus treatment in the final results of EXIST-1 and 2 [25, 26] or in the TOSCA Pass sub-study [12]. Embolisation because of flank pain was described in one patient under treatment in EXIST-2 [25].

### SEGA effectiveness

SEGA volume decreased in all three patients. Our observation (43–71% volume reduction) is in line with or better than results from the EXIST-1 trial, in which 35% [22] and 58% [26] of the patients had at least a 50% reduction in SEGA volume. This should, however, be interpreted with caution, as only three patients were included.

### Safety

The frequency of adverse effects was in line with EXIST-1–3 [24, 26, 53] but higher than reported in other studies [37, 39, 41, 47, 55]. Adverse effects ≥ grade 3 were reported in 35% of the patients, and were quite similar to those of EXIST-1 and 3 [22, 24], and varied between 0 and 35% in other studies [37, 46, 47, 55, 56].

Notably, patients with adverse effects ≥ grade 3 were younger than patients without adverse effects ≥ grade 3, this is in line with results in the extension study of EXIST-3 [27], making it especially important to be aware of management and follow up of adverse effects in younger patients.

The frequency of stomatitis/oral ulceration was slightly lower than those reported in EXIST-1–3 [22–24], and varied between 14 and 91% in other studies [37, 41, 46]. The study with the highest frequency reported a higher proportion of patients with intellectual disabilities and suggested that oral care might be insufficient [46].

The upper respiratory tract infections in our study were quite similar to those in EXIST-1–3 [22–24], but our frequency was higher than those reported in other studies [37, 55, 56]. Hypercholesterolemia, hypertriglyceridemia, and leukopenia were reported more frequently than in EXIST-1–3 [22–24, 26], and varies in other studies [29, 37, 46, 56]. Inequality in patient selection, serum concentration, management, education, and follow-up might explain some of the variation in the frequency and severity of adverse effects.

Dose reductions and interruptions were reported in 50%, as reported in EXIST-2 [53], and were higher than those reported in other studies (22–31%) [37, 41, 55]. Dose reductions or interruptions should be considered with adverse events ≥ grade 2 [57], and the high frequency in our study might reflect that management recommendations were followed [57]. However, dose reductions and interruptions occurred in only 25% of patients with stomatitis/oral ulcerations. For almost half of patients with symptomatic stomatitis/oral ulcerations everolimus treatment were not modified indicating that monitoring and management of adverse effects still needs to be better implemented.

Immunodeficiency disorder was described in one patient. This is a serious condition and is as far as we know not previously described as an everolimus related adverse event. Immunoglobulins were not measured before initiation of everolimus in this patient, and consequently it was not possible to determine for certain whether the patient that developed immunodeficiency disorder has a primary immunodeficiency, or if this is drug related. Clinically infections increased significantly after treatment initiation, making everolimus treatment a probable cause.

Data collection was finished for most patients before the outbreak of covid-19, only 14 had follow-up for days up to a month after the outbreak. For two of the nine patients that discontinued everolimus treatment, the COVID-19 pandemic was part of the reason, but apart from that it is not likely that the study result was influenced by the pandemic.

### Strengths and limitations

The study included unselected patients from two countries with similar health care systems and follow-up from specialists. The patients were recruited from the Norwegian TSC population and from two regions in Denmark. It is possible that some treated patients invited through the National Centre for Rare Epilepsy-Related Disorders in Norway did not respond to the invitation. Only two invited patients treated with everolimus did not want to participate. Due to equal access to health care services and follow-up from specialists, it is likely that most patients with indications for treatment are known and included. In Denmark, everolimus treatment is centralised, and all treated adult patients from Aarhus University Hospital and all paediatric patients from University Hospital Rigshospitalet were invited. The total response rate in Denmark was high (88%), indicating that the included patients were representative and the risk of selection bias low.

Due to the observational design, missing data in medical records and data unconformity were important limitations. Patients’ and parents’ interviews were carried out to reduce this limitation, but potential recall bias could not be excluded. Imaging data were not available for all patients and reduced the sample size. Imaging was reevaluated by experienced radiologists, ensuring data conformity.

Due to the irregular (multilobular) shape of most of the SEGAs, the simplified method of volume measurement by multiplying diameters in three orthogonal directions and divided the result by 2 was considered too inaccurate. Despite the chosen method, some inaccuracy in volume measurements may persist due to differences in imaging quality across multiple MRI exams from different centres and over the actual time period.

Other ASMs were changed in a majority of the patients, and some underwent epilepsy surgery, which made it difficult to draw conclusions on the cause of the change in seizure frequency. Different conditions for epilepsy treatment between Norway and Denmark were also limitations.

## Methods

### Patient selection and inclusion

Patients with a confirmed TSC diagnosis based on recommendations of the 2012 International TSC Consensus Conference [58] who were in current or previous treatment with everolimus were recruited from the registries of the Norwegian National Centre for Rare Epilepsy-Related Disorders, the Norwegian National Centre for Epilepsy, and the paediatric, renal, neurological, and rehabilitation departments in Norway, Aarhus University Hospital, and University Hospital Rigshospitalet, Copenhagen, Denmark between March 2019 and July 2020.

In Norway, 190 TSC patients from the registries were contacted about the use of everolimus: 75 replied, 35 of those were treated with everolimus and were included in the study. Two patients treated with everolimus did not consent to participate. Four were included from the renal, neurological, and rehabilitation departments. In Denmark, everolimus treatment is centralised. Among the users of everolimus, 25 were invited from Aarhus University Hospital, 20 of those were included, and 11 were invited from Rigshopitalet in Copenhagen, 9 of those were included.

#### Study design

This was a retrospective observational study. Demography, patient history, indication for and duration of treatment, dosage, serum concentration measurements, dose modifications/discontinuation and adverse effects, and seizure response in epilepsy patients were collected from medical records through a web-based form. Imaging data were re-evaluated by an experienced abdominal and neuro-radiologist. Data related to adverse effects and seizure responses in epilepsy patients were further assessed with a semi-structured patient/parent interview. Patients treated solely for lymphangiomyomatosis (LAM) were only included in the evaluation of adverse effects. In Denmark, the use of everolimus for epilepsy is restricted and requires 33% and 50% seizure reduction, good cooperation, and tolerable adverse effects after 4 and 12 months respectively for permission to continue treatment. To fulfil these requirements, Danish patients submit seizure diaries for review, and the treating physician submits an evaluation to the Danish Medicines Authorities for documentation after the first treatment year. Patients are not obliged to submit seizure registrations in Norway.

#### Outcome measures of epilepsy

We investigated changes in seizure frequency from baseline to the last three months of everolimus treatment. We defined seizure frequency during the last three months before treatment as the baseline. Seizure response was divided into seizure freedom, ≥ 50% reduction, ≥ 30% reduction, clinically relevant reduction without a given percentage (when percentage change was not available), no change, or increase. The total reduction in the frequency of focal, tonic, myoclonic, atonic, and focal to bilateral tonic clonic seizures was calculated. Atypical absences were not included. Effectiveness was described in three groups: (a) all everolimus patients with epilepsy using anti-seizure medication (ASM), with ≥ 1 seizure/year (entire epilepsy group), (b) patients with epilepsy treated with everolimus for other indications (other indications group), and (c) patients treated with everolimus for epilepsy indication (epilepsy indication group). Changes in ASM treatment were described as none, minor (dosage adjustments, discontinuing an ASM), or major (adding an ASM, vagus nerve stimulator, or epilepsy surgery).

We further investigated the association between epilepsy effectiveness and epilepsy severity, age, and concentration-to-dose ratio (C/D ratio) as a measure of drug exposure. The C/D ratio was calculated from the mean serum concentration/dose in patients with at least three everolimus measurements. Epilepsy severity was investigated by association between the number of seizure types (< 3, ≥ 3), occurrence of focal to bilateral tonic clonic seizures, median weekly seizure frequency (< 7, ≥ 7 in the entire epilepsy group, < 1.5, ≥ 1.5 in the other indication group and < 25, ≥ 25 in the epilepsy indication group), and number of ASMs (< 3, ≥ 3).

#### Outcome measures of RAML and SEGA

We investigated the change in the size of rAML (largest lesion and mean diameter change of largest lesion in both kidneys) from baseline to the last imaging and the change in SEGA volume from baseline to the last imaging. RAMLs with the longest diameter (LD) in both kidneys and overall were identified. The change in size was calculated by subtracting the LD at baseline from the LD at the last imaging. The baseline was defined as imaging closest to the start of treatment. The change in size was defined according to RECIST [54], with progression defined as at least 20% increase, response as at least 30% decrease, and stable size between 20% increase and 30% decrease in LD.

SEGA volume was calculated by manually drawing areas along tumour borders on every axial slice (1 mm slice thickness), summing areas and multiplying the result with the slice interval (usually 1 mm), performed on a PACS workstation (SECTRA PACS software). For every exam, a T1-weighted isotropic volume series (MPRAGE) was used, contrast enhanced when available, and alternatively without contrast.

The effectiveness of everolimus treatment for rAML (> 1 cm) and SEGA lesions was investigated when imaging was available and when everolimus was prescribed for these indications. The quality of renal imaging was defined as good, moderate, or poor.

We further investigated the change in the number of rAML > 1 cm and renal complications (haemorrhage, embolization, nephrectomy) during everolimus treatment.

#### Outcome measures of adverse effects

We investigated adverse effects possibly or probably related to everolimus treatment mapped and graded by the National Cancer Institute Common Terminology Criteria version 5 [59] over time (1st, 2nd, after 2nd treatment year, and throughout the entire treatment period). The grading refers to a clinical description of severity of the adverse effects divided into following grading: mild, moderate, severe, life treating and death [59].

Hypercholesterolemia was not graded using this terminology. Grading was based on total cholesterol levels (grade 1: increased from start or under treatment to above the upper limit of normal (ULN) to 7.75 mmol/L, grade 2: 7.75–10.34 mmol/L, grade 3: 10.34–12 mmol/L). Adverse effects were described every year they occurred. Adverse effects without information of date and without information of grade were described and included in the entire treatment period. We further investigated dose modifications (interruptions, and dose reductions), discontinuation, reason for discontinuation, and association between adverse effects ≥ grade 3 and age.

#### Statistical analysis

The data were coded and analysed with SPSS (version 28.0.1.1 (14)). Continuous variables were analysed with frequency, mean, standard deviation, median, minimum, and maximum. Contingency tables with chi-square test for independence (Pearson’s chi-square and Fisher’s exact test) were used to test group differences between categorical variables (seizure reduction and related factors (≥ 3/< 3 seizure types, GTK, ≥ 3/< 3 ASMs, median weekly seizure frequency, age at start of treatment ≥ 18/< 18, major change in ASMs, and occurrence of adverse effects). Group differences in rAML, seizure reduction, frequency of adverse effects, and grade 3–4 adverse effects between Norway and Denmark were analysed by contingency tables with chi-square test for independence (Pearson’s chi-square and Fisher’s exact test). The Mann–Whitney U test was used to test group differences between continuous not normally distributed variables, occurrence of grade 3–4 adverse events, and group differences between CD/ratio and seizure reduction/rAML reduction. A *p* < 0.05 was considered statistically significant.

## Conclusions

The effectiveness of everolimus in epilepsy was acceptable and in line with EXIST-3. In this study, however, most patients also changed their concomitant ASM treatments. This may suggest that everolimus was not the only cause of the improvement in the seizure situation. Treatment effectiveness with everolimus was associated with a younger age. The results indicate that everolimus treatment reduces or stabilises rAML lesions, reduces risk of renal events, and reduces SEGA volume and risk of hydrocephalus.

Most adverse effects were generally mild to moderate, but some tended to be more frequent than in EXIST and other clinical practice studies. Careful monitoring of adverse effects is needed, and benefits against adverse effects should be carefully considered and discussed with patients and parents before the start of treatment and during follow-up. It is our opinion that awareness in vulnerable patients, such as children and patients with intellectual disabilities and autism disorders, is of special importance.

### Supplementary Information

**Additional file 1: Table S1.** Seizure reduction versus no seizure reduction and related factors. **Table S2.** Seizure reduction (≥/<30 %) and related factors.

## Data Availability

The datasets generated during and/or analysed during the current study are not publicly available due to them containing information that could compromise research participant privacy/consent but are available from the corresponding author on reasonable request.

## Abbreviations

TSC: Tuberous sclerosis complex
LD: Longest diameter
RECIST: Response evaluation criteria in solid tumors
m-TOR: Mammalian target of rapamycin
rAML: Renal angiomyolipoma
SEGA: Subependymal giant cell astrocytomas
ASM: Anti-seizure medication
C/D ratio: Concentration-to-dose ratio
