# Supplementary material for: Effectiveness and safety of everolimus treatment in patients with tuberous sclerosis complex in real-world clinical practice
Source: Orphanet J Rare Dis. 2023 Dec 2;18:377. doi: 10.1186/s13023-023-02982-1 (PMC10693167; doi:10.1186/s13023-023-02982-1)
Supplement: Supplementary file 1 — Additional file 1: Table S1. Seizure reduction versus no seizure reduction and related factors. Table S2. Seizure reduction (≥/<30 %) and related factors. [file 13023_2023_2982_MOESM1_ESM.docx]

| **Additional file 1: Table S1. Seizure reduction versus no seizure reduction and related factors** | | | | | | | | | |
| --- | --- | --- | --- | --- | --- | --- | --- | --- | --- |
|  |  | **Entire epilepsy group**  **(n = 45)** | | **Other indications group**  **(n = 17)** | | |  | **Epilepsy indication group**  **(n = 28)** | |
|  |  | Seizure reduction  n/N/ % | No seizure reduction  n/N (%) |  | Seizure reduction  n/N/ % | No seizure reduction  n/N (%) |  | Seizure  reduction  n/N/ % | No seizure  reduction  n/N (%) |
| All  Norway  Denmark |  | 26/45 (58)  15/26 (58)  11/19 (58) | 19/45 (42)  8/26 (42)  11/19 (42) |  | 10/17 (59)  5/7 (71)  5/10 (50) | 7/17 (41)  2/7 (29)  5/10 (50 |  | 19/28 (68)  13/19 (68)  6/9 (67) | 8/20 (32)  6/19 (32)  3/9 (33) |
| ≥3 seizure types before treatment | Yes  No | 5/10 (50)  15/35 (43) | 5/10 (50)  20/35 (57) |  | 1/4 (25)  6/13 (46) | 3/4 (75)  7/13 (54) |  | 4/6 (67)  11/22(50) | 2/6 (33)  11/22 (50) |
| GTK before treatment | Yes  No | 11/22 (50)  15/23 (65) | 11/22 (50)  8/23 (35) |  | 3/10 (30)  4/7 (57) | 7/10 (70)  3/7 (43) |  | 3/4 (75)  12/24 (50) | 1/4 (25)  12/24 (50) |
| ≥3 ASM at start of treatment | Yes  No | 10/20 (50)  10/25 (40) | 10/20 (50)  15/25 (60) |  | 4/6 (67)  3/12 (25) | 2 (33)  8/12 (75) |  | 8/14 (53)  7/14 (50) | 6/14 (47)  7/14 (50) |
| Median weekly seizure  frequency before treatment** | <7  ≥ 7 | 6/15 (40)  14/26 (54) | 9/15 (60)  12/26 (46) | < 1.5  ≥ 1.5 | 2/5 (40)  5/10 (50) | 3/5 (60)  5/10 (50) | < 28  ≥ 28 | 6/13 (46)  9/13 (69) | 7/13 (54)  4/13 (31) |
| Age at start of treatment | <18  ≥ 18 | 17/22 (77)  9/23 (39) | 5/22 (23)*  14/23 (61) |  | 2/3 (67)  5/14 (36) | 1 (33)  9/14 (64) |  | 13/21 (62)  2/7 (29) | 8/21 (38)  5/7 (71) |
| Major change in ASMs | Yes  No | 11/19 (58)  15/26 (58) | 8/19 (42)  11/26 (42) |  | 4/7 (57)  3/10 (30) | 3/7 (43)  7/10 (70 |  | 7/12 (58)  12/16 (75) | 5/12 (42)  4/16 (25) |

*Significant difference

** Patients with information

| **Additional file 1: Table S2.: Seizure reduction (≥/<30 %) and related factors** | | | | | | | | | |
| --- | --- | --- | --- | --- | --- | --- | --- | --- | --- |
|  |  | **Entire epilepsy group**  **(n = 45)** | | **Other indications group**  **(n = 17)** | | | **Epilepsy indication group**  **(n = 28)** | | |
|  |  | ≥ 30 % seizure reduction  n/N/ % | < 30% seizure reduction  n/N(%) |  | ≥ 30 % seizure reduction  n/N/ % | < 30% seizure reduction  n/N(%) |  | ≥ 30% seizure reduction  n/N(%) | < 30 % seizure reduction  n/N (%) |
| All  Norway  Denmark |  | 20/45 (44)  10 /26 (38) 10/19 (53) | 25/45 (56)  16/26 (62)  9/19 (47) |  | 5/17 (29)  1/7 (14)  4/10 (40) | 12/17 (71)  6/7 (86)  6/10 (60 |  | 15/28 (54)  9/19(47)  6/9 (67) | 13/28 (46)  10/19 (53)  3/9 (33) |
| ≥ 3 seizure types before treatment | Yes  No | 5/10 (50)  15/35(43) | 5/10 (50)  20/35(57) |  | 1/4 (25)  4/13 (31) | 3/4 (75)  9/13 (69) |  | 4/6 (67)  11/22(50) | 2/6 (33)  11/22 (50) |
| GTK before treatment | Yes  No | 8/22 (37)  12/23 (52) | 14/22 (64)  11/23 (48) |  | 3/10 (30)  2/7 (29) | 7/10 (70)  5/7 (71) |  | 3/4 (75)  12/24(50) | 1/4 (25)  12/24 (50) |
| ≥ 3 ASM at start of treatment | Yes  No | 10/20 (50)  10/25 (40) | 10/20(50)  15/25 (60) |  | 2/5 (40)  3/12 (25) | 3/5 (60)  9/12 (75) |  | 8/14(53)  7/14(50) | 6/14(47)  7/14(50) |
| Median weekly seizure**  frequency before treatment | <7  ≥ 7 | 6/15 (40)  14/26 (54) | 9/15(60)  12/26 (46) | < 1.5  ≥ 1.5 | 1/7 (14)  4/8 (50) | 6/7 (86)  4/8 (50) | < 28  ≥ 28 | 6/13(46)  9/13(69) | 7/13(54)  4/13(31) |
| Age at start of treatment | <18  ≥ 18 | 15/24 (63)  5/21 (24) | 9/24 (38)*  16/21 (76) |  | 2/3 (67)  3/14 (21) | 1/3 (33)  11/14 (79) |  | 13/21(62)  2/7(29) | 8/21(38)  5/7(71) |
| Major change in ASMs | No  Yes | 12/26 (46)  8/19 (42) | 14/26 (54)  11/19 (58) |  | 3/10 (30)  2/7 (29) | 7/10 (70)  5/7 (71) |  | 9/16(56)  6/12(50) | 7/16(44)  6/12(50) |
| *Significant difference  ** Patients with information | | | | | | | | | |
